# A Case Report of Behcet's Disease With Thromboses in the Superior Vena Cava and External Iliac Vein

**DOI:** 10.7759/cureus.24064

**Published:** 2022-04-12

**Authors:** Faiza Zafar Sayeed, Muhammad Nashit, Shaheen Bhatty, Bushra Z Sayeed, Fariha Asad

**Affiliations:** 1 Department of Internal Medicine, Dr. Ruth K. M. Pfau Civil Hospital Karachi, Karachi, PAK

**Keywords:** mucocutaneous ulcers, pathergy, cardiovascular disease, thrombotic angiopathy, autoimmune disease, thromboembolic disease, behcet's syndrome, superior vena cava (svc) syndrome, behcet's disease

## Abstract

Behcet's disease (BD), also known as Behcet's syndrome, is a rare, chronic, autoimmune disorder of unknown origin. Its manifestations are thought to be caused by vasculitis, resulting in damage to blood vessels of all sizes throughout the body. We report a 25-year-old Pakistani male who is sexually active and presents with a one-year history of shortness of breath, cough, exertional dyspnea, and neck and facial swelling. On examination, he had severe anemia, mouth ulcers, distended neck and chest veins, prominent abdominal veins, and a scrotal ulcer. After going through mandatory investigations to evaluate the presenting signs and symptoms, thromboses were found in the major veins, including the superior vena cava (SVC) and external iliac vein, as well as a positive pathergy test. Accordingly, a diagnosis of Behcet’s disease with cardiomyopathy and venous thrombosis was made. He was treated with anticoagulants, steroids, and azathioprine for six months and subsequently went into remission.

## Introduction

Behcet’s disease (BD) is a multisystem disorder having a relapsing and remitting course, characteristically manifesting with mucocutaneous, ocular, and gastrointestinal symptoms. Most of the cases have been reported from Turkey. Current literature shows that BD can present as a myriad of symptoms with the involvement of multiple organ systems, including skin, urogenital, neurological, and vascular, among others [1]. Here, we highlight a case that presented with cardiovascular involvement and thrombotic complications, which are known to be rare manifestations of BD. A retrospective study comprising 5970 patients showed vascular involvement in 14.7% of cases [2]. Venous system involvement is far more common than the arterial system, with literature proving so in up to 10-25% of patients [3]. Nonetheless, a classic presentation of the disease would involve recurrent oral and genital ulceration with uveitis or any other ocular symptom [4].

Fei et al. [3] and Lee et al. [5] report multiple kinds of thrombosis in BS patients, with DVT being the most common. Other thromboses reported were the superior vena cava (SVC), the pulmonary artery, and a few cases presenting as intracardiac thrombus. External iliac vein thrombosis, on the other hand, is considered a rare manifestation with only a few instances found in the current literature, especially in the absence of coagulation abnormalities [1,6]. Furthermore, BD is very rarely reported from this part of the world in Southeast Asia, making our case unique in the medical literature.

We report the case of a young male with recurrent aphthous and genital ulcers, facial swelling due to thrombosis of the superior vena cava, and an incidental finding of external iliac vein thrombosis on CT as the initial presentation of Behcet’s disease.

## Case presentation

**Figure 1:**
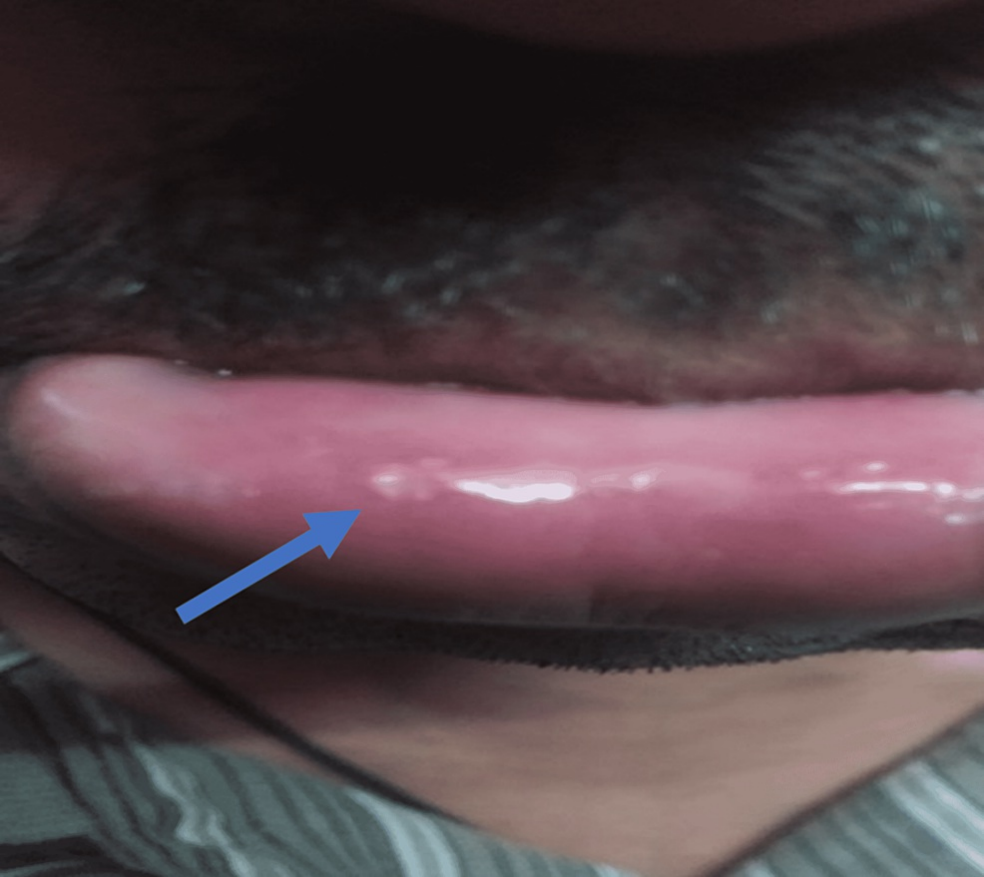
Aphthous ulcer on lower lip

**Figure 2:**
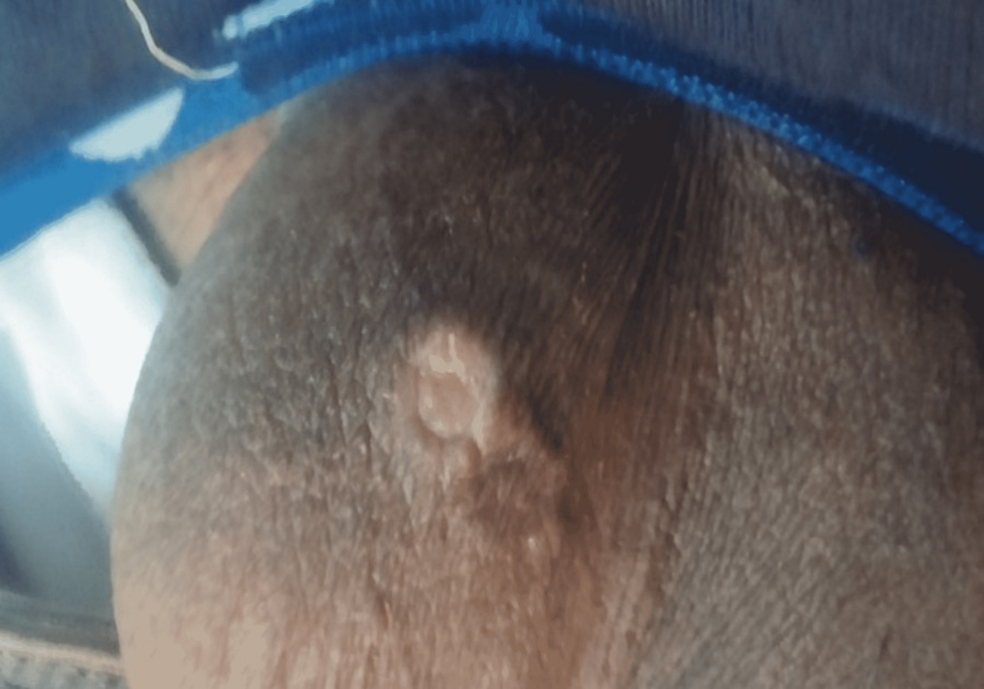
Ulcer seen on scrotum

A 25-year-old Pakistani male presented to us with a history of dry cough, dyspnea, and facial edema for one year, with rapid progression of symptoms in the past six months. On clinical examination, the patient appeared pale with facial swelling, an engorged neck, abdominal veins, and aphthous ulcers on the lower lip and scrotum (Figures 1-2).

Fundoscopy revealed small hemorrhages in the retina of the left eye. Laboratory investigations revealed anemia with hemoglobin of 10.1 g/dl, hematocrit of 33%, mean corpuscular volume of 73 fl, c-reactive protein (CRP) of 111 mg/l, erythrocyte sedimentation rate (ESR) of 104 mm/h, international normalized ratio (INR) of 1.33, and all other investigations, including liver and renal function, electrolytes, protein levels, viral markers, thyroid and thrombophilia profiles, venereal disease research laboratory test (VDRL), HIV RNA, anti-nuclear antibody, anti-double-stranded DNA, and lipid profiles, were reportedly normal (Table 1). Urinalysis was also not significant (Table 2). Urine and blood cultures revealed no growth.

**Table 1:** Laboratory investigations ALP: alkaline phosphatase, ALT: alanine transaminase, ANA: antinuclear antibody, Anti-ds DNA: anti-double-stranded DNA, AST: aspartate transaminase, GGT: gamma-glutamyl transferase, HDL: high-density lipoprotein, LDL: low-density lipoprotein, VDRL: venereal disease research laboratory test

Investigations	Values	Reference range
Complete blood count		
Hemoglobin	10.1 g/dl	13–16 g/dl (males)
Hematocrit	33%	38–48% (males)
Mean corpuscular volume	73 fl	80–100 fl
White blood cells	9200/µl	4000–10,000/µl
Platelets	307,000/µl	150,000–450,000/µl
Renal function tests		
Blood urea nitrogen	22 mg/dl	6–24 mg/dl
Creatinine	0.7	0.7–1.3 mg/dl (males)
Sodium (Na^+^)	137 mEq/l	135–145 mEq/l
Potassium (K^+^)	3.8 mEq/l	3.6–5.2 mEq/l
Chloride (Cl^-^)	98 mEq/l	96–106 mEq/l
Bicarbonate ($\textrm{HCO}_{3}^{-}$)	22 mEq/l	22–29 mEq/l
Thrombophilia profile		
Prothrombin time	16.7 s	11–13.5 s
Activated partial thromboplastin time	35 s	21–35 s
International normalized ratio	1.33	0.8–1.1
Liver function tests		
Total bilirubin	0.72 mg/dl	0.1–1.2 mg/dl
ALT	36 IU/l	7–55 IU/l
AST	34 IU/l	8–48 IU/l
ALP	77 IU/l	44–147 IU/l
GGT	27 IU/l	0–30 IU/l
Lipid profile		
Triglyceride	135 mg/dl	<150 mg/dl
HDL	65 mg/dl	45-70 mg/dl
LDL	75 mg/dl	<100 mg/dl
Viral markers		
Hepatitis B surface antigen	Negative	
Hepatitis C antibody	Negative	
Other tests		
Total protein	7.0 g/dl	6.0-8.3 g/dl
Albumin	3.7 g/dl	3.4-5.4 g/dl
Globulin	3.0 g/dl	2.0-3.9 g/dl
Albumin/globulin ratio	1.5	1.1-2.5
Erythrocyte sedimentation rate	111 mm/h	1-13 mm/h (males)
C-reactive protein	104 mg/l	8-10 mg/l
Human immunodeficiency virus, antigen/antibody	Non-reactive	
ANA	Negative	
Anti-ds DNA	Negative	
VDRL	Negative	

**Table 2:** Urine detailed report

Urine detailed report	
Color	Dark yellow
Appearance	Clear
Specific gravity	1.030
pH	6.0
Protein	Negative
Glucose	Negative
Ketones	Negative
Red blood cells	Nil/hpf
White blood cells	1/hpf
Casts	Nil
Nitrites	Negative
Leukocyte esterase	Negative
Epithelial cells	1/hpf
Crystals	Nil

**Figure 3:**
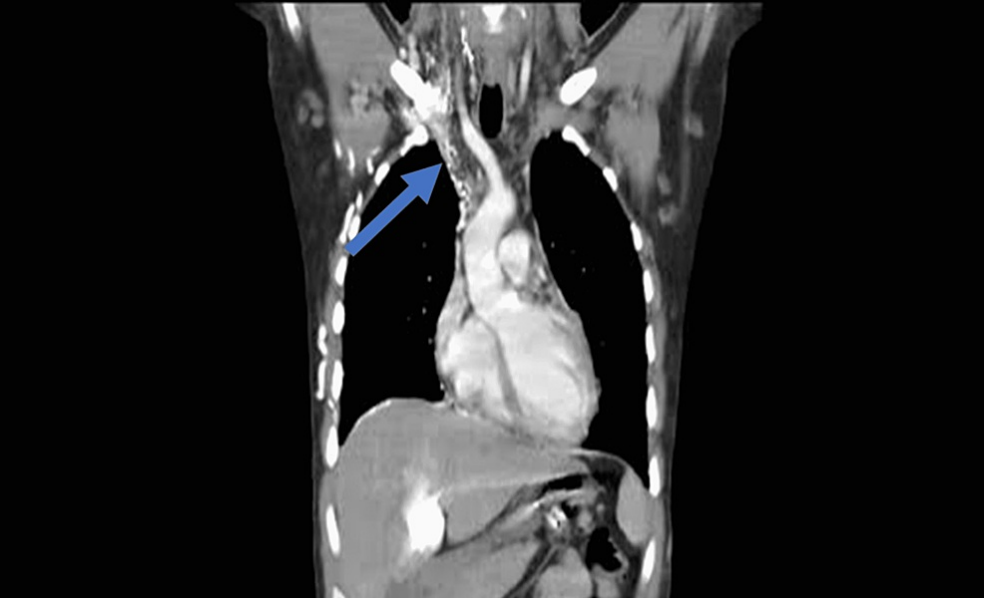
CT chest showing SVC thrombosis SVC: superior vena cava

The chest X-ray was unremarkable with no consolidation, opacifications, or prominent markings in the lobes, clear costophrenic angles, normal lung cavity size, and cardiac shadow. The ECG showed low voltage with non-specific ST changes, excluding a cardiac electrophysiological pathology. He had echocardiography done, which revealed generalized hypokinesia of the left ventricle without an intramural thrombus, an ejection fraction of 35%, and normal sizes of the heart chambers. In light of the aforementioned investigations not leading to a specific diagnosis and an echocardiography report showing severe cardiac dysfunction, a CT scan of the chest and abdomen was ordered, which showed superior vena cava thrombosis (Figure 3). 

**Figure 4:**
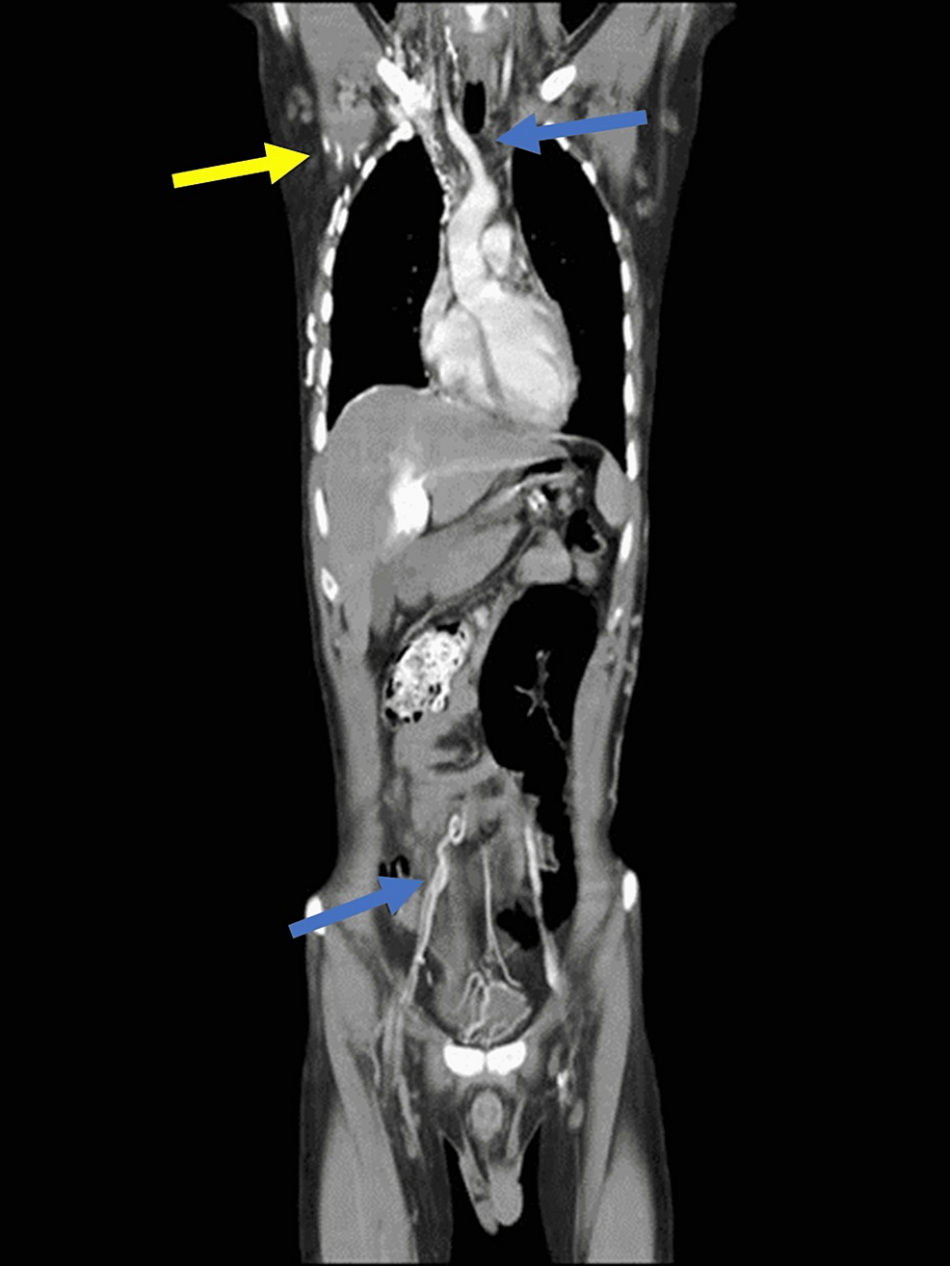
CT CAP showing SVC and right external iliac vein thromboses (blue arrows) and collaterals in axilla (yellow arrow) CT CAP: computed tomography chest abdomen pelvis, SVC: superior vena cava

**Figure 5:**
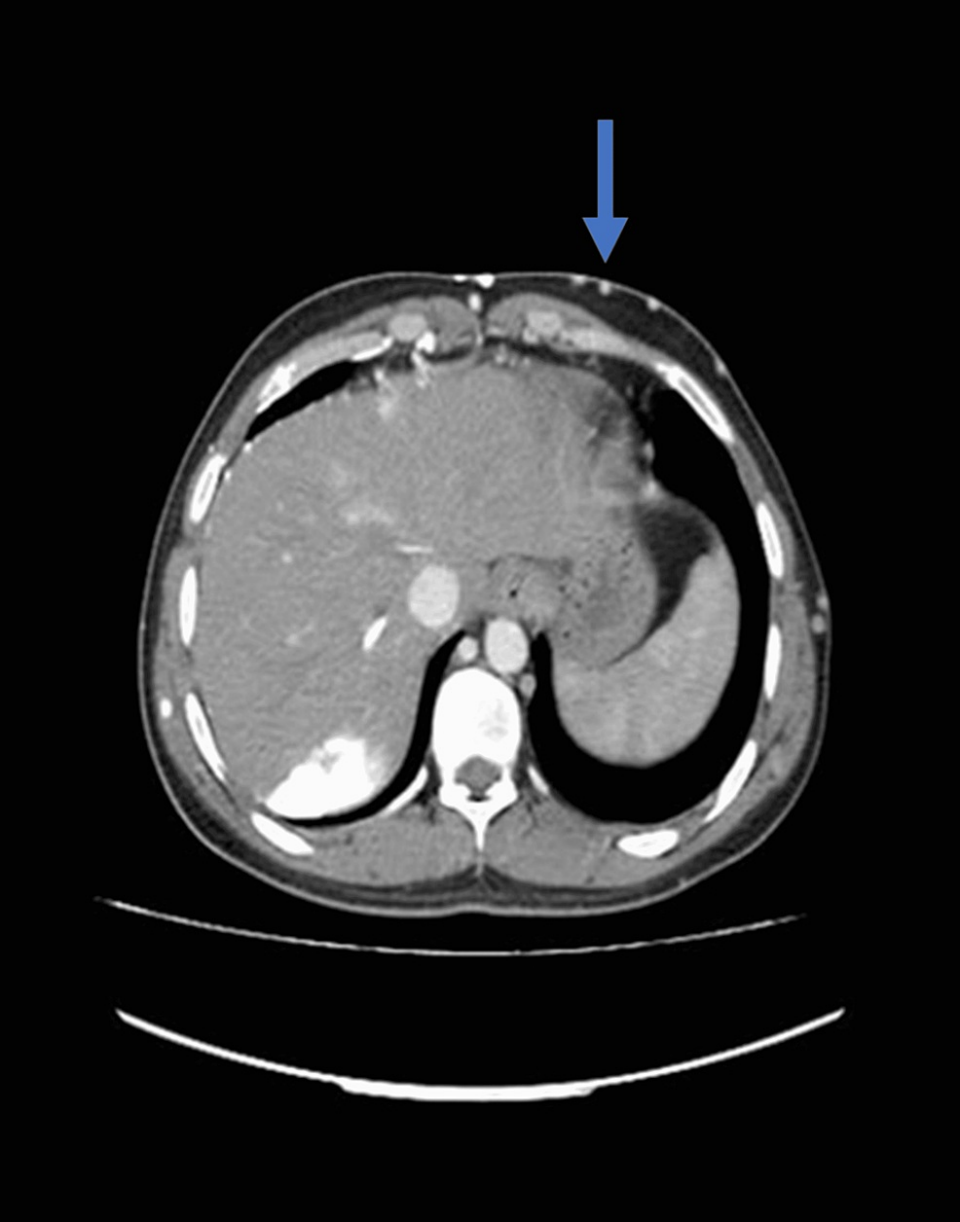
CT scan abdomen showing abdominal wall collaterals CT: computed tomography

It also revealed a thrombus in the right external iliac vein as well as multiple collaterals present in the axilla and abdominal wall (Figures 4-5).

As he had raised inflammatory markers with thrombosis in major veins, severe cardiac dysfunction with a normal thrombophilia profile, and recurrent mouth ulcers and genital ulcers, a diagnosis of Behcet’s disease was made, and a pathergy test was performed, resulting in a pustule on the injection site, strengthening our diagnosis of BD. Hence, our patient presented to us with BD with underlying cardiovascular symptoms.

He was started on a high-dose of methylprednisolone (1 g/day) for five days before switching to prednisone (60 mg/day P.O.). The patient was seen to improve clinically, also evidenced by an improvement in repeat inflammatory markers. Furosemide was also instituted with regards to a low ejection fraction (35%) on echocardiography. Concomitantly, he was started on Azathioprine (100 mg/day P.O.) and Warfarin for anticoagulation to maintain an INR of 2.5. No further complications were found during a six-month follow-up, and he was found to be in remission.

## Discussion

According to the International Study Group criteria for Behcet’s syndrome (1990), it is stated that for a diagnosis of Behcet’s syndrome to be made, there must be recurrent mouth ulcers present along with any two of the following: genital ulcers, eye lesions, skin lesions, or a positive pathergy test [7]. Our patient had recurrent ulceration and positive fundoscopy findings. Thus, keeping his sexual history in consideration, he was investigated for syphilis and HIV, which came out to be unremarkable. On CT chest and abdomen, he was found to have thrombosis of the superior vena cava and external iliac vein with multiple collaterals.

He was started on diuretics, steroids, anticoagulants, and immunomodulatory agents, which showed improvement in symptoms. Patients with Behcet’s disease show pathergy reactions, which is a phenomenon in which the skin induces an aggravated response towards injury, resulting in a follicle.

A study conducted in Iran on COVID-19 patients having Behcet’s disease reported that 63% of cases showed pathergy phenomenon [8]. These lesions differ from the other ulcers seen in the disease on skin and mucosa. Pathergy is also seen in a waxing and waning pattern [9].

Venous system abnormalities are significantly more common than those of the arterial system. Behcet’s disease is known to involve the major vessels and cause DVT (deep venous thrombosis) in many patients [10].

Ranging from the most common to least common manifestations seen in this disease are oral ulcers, genital ulcers, erythema nodosum, gastrointestinal lesions, fever, arthralgias, ocular lesions, and vascular involvement [11].

Fei et al. conducted a retrospective study on 796 patients, of which 20.6% had cardiac involvement along with vascular involvement. Cardiac involvement includes pericarditis, myocarditis, endocarditis, and ventricular and valvular pathologies [3].

The pathophysiology of venous thrombi as seen in our patient is presumably due to the thickening of the vessel lumen due to inflammation. These are not the usual thromboses made of plaques, and hence embolization is not seen. This is also thought to be the reason for the very few small vessel symptoms reported in this disease [12].

## Conclusions

Immunosuppressive drugs are the cornerstone of treatment for Behcet’s disease and, more importantly, for those with vascular symptoms. A definitive cure is yet to be discovered, but for now, patients are to be well informed of the relapsing and remitting course of the condition and required to follow through with the treatment regimen. We managed our patient with anticoagulants with close monitoring of INR, diuretics, steroids, and immunosuppressive agents.
